# Salidroside impedes Ang II-infused myocardial fibrosis by activating the SIRT1-Nrf2 pathway

**DOI:** 10.22038/ijbms.2025.83659.18105

**Published:** 2025

**Authors:** Xi Zhu, Zhen Hai, Zhongping Ning

**Affiliations:** Department of Cardiology, Shanghai Pudong New Area Zhoupu Hospital (Shanghai Health Medical College Affiliated Zhoupu Hospital) , Shanghai 201318, China

**Keywords:** Angiotensin II, SIRT1, Oxidative stress, Reactive oxygen species, Salidroside

## Abstract

**Objective(s):**

This research examined the protective function of salidroside (SAL) against angiotensin II (Ang II)-infused myocardial fibrosis and its associated mechanism.

**Materials and Methods:**

The C57BL/6 male murine models (n=24) received either saline solution or Ang II (1500 ng/kg/day) subcutaneously and an oral dosage of SAL (50 mg/kg/day) once daily for 28 days. Newborn Sprague-Dawley (SD) rats were used to isolate atrial fibroblasts.

**Results:**

The fibrotic region was raised by Ang II infusion, while SAL treatment inhibited it. Collagen I and III expression was raised by Ang II induction, but SAL therapy reduced their expression. SAL therapy also decreased the expression of other fibroblast differentiation-related markers induced by Ang II infusion. It elevated SIRT1, Nrf2, and HO-1 levels in atrial fibroblasts. Additionally, SAL significantly inhibited atrial fibroblasts, whereas EX527, an inhibitor of SIRT1, noticeably increased the migration ability. Furthermore, SAL suppressed intracellular ROS production and oxidative stress in Ang II-infused atrial fibroblasts.

**Conclusion:**

SAL protects against myocardial fibrosis infused by Ang II by activating the SIRT1-Nrf2 pathway.

## Introduction

Various pathophysiological processes, such as valvular heart disease, hypertension, and myocardial ischemic injury, cause myocardial fibrosis. Unusual collagen accumulation in the cardiac muscle, resulting from improper macrophage migration and myofibroblast proliferation, is frequently considered (1). When this mechanism becomes hyperactive, it causes left ventricular hypertrophy and reduced LV function (2). Clinical investigations have found a link between ventricular fibrosis, LV hypertrophy, cardiac attack, and other cardiovascular disorders in hypertensive patients (3). Therefore, identifying novel regulators of myocardial fibrosis is urgently needed to improve effective treatment options for myocardial fibrosis.

The abnormal stimulation of the renin-angiotensin system (RAS) significantly impacts the progression of certain cardiovascular conditions. The RAS’s principal effector peptide is referred to as angiotensin II (Ang II). It is synthesized within the circulatory system and localized cardiac tissues. Ang II plays a substantial role in the improvement of several cardiac conditions, such as myocardial infarction, diabetic cardiomyopathy, and alcoholic cardiomyopathy (4-6). Reactive oxygen species (ROS) are produced when Ang II binds to its receptor, AT1, which, in turn, initiates the stimulation of nicotinamide adenine dinucleotide phosphate (NADPH) oxidase (7). Oxidative stress is caused by the excessive production of ROS that exceeds the body’s capacity to scavenge them. Ang II infusion induces oxidative stress, promptly activating the apoptotic signaling pathway. Damage to cardiomyocytes can result in necrosis or apoptosis, which may lead to remodeling of the ventricular and, ultimately, cardiac arrest (8). 

Additionally, excessive ROS can induce myocardial inflammation, apoptosis, ventricular remodeling, hypotrophy, and myocardial inflammation through the receptors for nuclear factor-kappa B (NF-kB), mitogen-activated protein kinase, and epidermal growth factor (9, 10). A class III histone deacetylase that relies on NAD+, SIRT1, is essential in regulating physiological responses to oxidative stress, metabolic processes, and inflammatory disorders by suppressing proinflammatory cytokine production (11). A previous investigation found that stimulating SIRT1 might lessen the inflammatory reaction triggered by sepsis and protect against acute renal damage (12). Early research reported that the Nrf2 signal channel controls system Xc_¯_ activity and expression (13). In addition, SIRT1 activates Nrf2, which improves anti-oxidant defenses and protects against Ang II-infused oxidative stress (14). However, the SIRT1/Nrf2 pathway functions as a balancing mechanism, increasing anti-oxidant responses while inhibiting inflammation, and it is affected by Ang II signaling (14, 15). Therefore, modulating the SIRT1/Nrf2 pathway might be a promising strategy for inhibiting Ang II-induced cardiac fibrosis.

**Figure 1:**
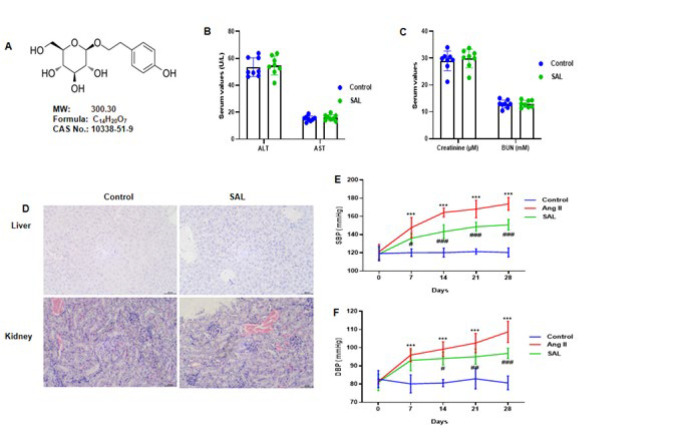
The study investigated the* in vivo* tolerance of salidroside in mice

Salidroside (SAL), discovered in Rhodiola, is known as a tyrosol glycoside, which is a medicinal compound due to its depression medication and antianxiety properties (16). Figure 1 represents the chemical structure of SAL. SAL has recently received increased interest owing to its many medicinal effects, which include anti-oxidants, anti-inflammatory properties, cancer prevention, and neural protection (17-19). SAL has decreased severe lung injury in mice exposed to LPS or paraquat (20). In addition, SAL prevents sepsis and LPS-infused severe lung damage in mice (21). However, SAL’s mechanism and protective function against Ang II-infused myocardial fibrosis have not yet been explored. Therefore, the research aimed to explore the associated molecular mechanisms and the potential therapeutic benefits of SAL in reducing Ang II-induced cardiac fibrosis.

## Materials and Methods

### Animals and treatment

A temperature-controlled, specified pathogen-free (SPF) environment, with a 12-hour light/dark phase and unrestricted entree to food and drink, was maintained for 24 male C57BL/6 mice. For 28 days, mice were subcutaneously administered an equivalent amount of PBS or Ang II (1500 ng/kg/day, HY-13948, MedChemExpress) using an Alzet osmotic minipump (Model 2004, USA). Mice were gavaged with SAL (50 mg/kg/day, HY-N0109, MedChemExpress) or an equal amount of PBS once a day for 28 days. For hepatic and renal toxicity experiments, mice were subdivided into (1) the PBS gavage group and (2) the SAL gavage group. For experiments involving the effects of salidroside, mice were categorized into three distinct categories: (1) the group receiving PBS infusion, (2) the group subjected to Ang II + PBS, and (3) the group subjected to Ang II + SAL, with a sample size of 8 for each group. On the other hand, the control group involved mice that were administered PBS via implanted Alzet minipumps and orally gavaged with PBS. Before the mice were sacrificed, they were anesthetized with an intraperitoneal dosage of sodium pentobarbital (50 mg/kg). The Zhoupu Hospital Ethics Committee, affiliated with Shanghai University of Medicine and Health Science, granted ethical approval for this research under reference number 2023-C-039-E01. The authors ensured adherence to all standard procedures outlined in the 1964 Declaration of Helsinki. The techniques employed in this investigation adhered closely to the recommendations of the ARRIVE protocol.

### Blood pressure measurement

The CODA-MNTR tail-cuff device from CODA, USA, was used to monitor the diastolic blood pressure (DBP) and systolic blood pressure (SBP) (22). Readings were taken the day before Ang II infusion and at 0, 7, 14, 21, and 28 days post-infusion. At 15:00, blood pressure was taken by the same people. Average results were obtained by measuring the SBP and DBP five times.

### Echocardiography

Two-dimensional echocardiography was conducted utilizing a Small Animal Ultrasound Imaging System (Vevo3100, VisualSonics, Canada). Anesthesia for mice was induced through inhaled isoflurane using a vaporizer and sustained at 1% isoflurane. The heart rate, left atrial diameter (LAD), left ventricular end-diastolic posterior wall thickness (LVPWth), ejection fraction (EF), and fractional shortening (FS) were measured using M-mode (23).

### Measurement of mouse heart weight (HW)

The mouse heart weight was measured using the previously described method (24). Twenty-eight days following Ang II infusion, each mouse’s body weight (BW), heart weight (HW), and tibia length (TL) were gauged. Subsequently, the HW/BW and HW/TL ratios were computed.

### Biochemical analysis

On the 28th day after the initial infusion of Angiotensin II, serum specimens were collected from the murine subjects to analyze biochemical indicators, encompassing the activities of alanine aminotransferase (ALT; C009-2-1, Nanjing Jiancheng Bioengineering Institute, Nanjing, China), aspartate aminotransferase (AST; C010-2-1, Nanjing Jiancheng Bioengineering Institute, Nanjing, China), and lactate dehydrogenase (LDH; E-BC-K046-M, Elabscience, China) (24).

### Enzyme-linked immunosorbent assay (ELISA)

On day 28, following the initial infusion of Ang II, blood serum was collected from mice to measure the concentrations of several cardiac biomarkers. These comprised cardiac troponin I (cTnI) (SEKM-0153, Solarbio, China), cardiac troponin T (cTnT) (SEKM-0150, Solarbio, China), CK-MB (SEKM-0152, Solarbio, China), ANP (E-EL-M0166c, Elabscience, China), and BNP (E-EL-M0204c, Elabscience, China). The measurements were conducted using ELISA kits. In order to calculate concentrations, a microplate reader used the standard curve to detect absorbance at 450 nm (25).

### Histology

Following overnight fixation in 4% paraformaldehyde, the liver, kidney, and heart were paraffin-embedded and sectioned at 5 µm. Hematoxylin and eosin (H&E) labeling was used to assess the morphology of the kidney and liver. The extent of collagen deposition in the left ventricle was determined using Masson’s trichrome staining. At least five arbitrary arenas of assessment were chosen from each section, and the area of fibrosis ratio was computed (26). Image-Pro Plus 6.0 was utilized to analyze the images.

### Isolation and culture of rat atrial fibroblasts

The ventricles of the 20 neonatal Sprague-Dawley (SD) rats were dissected and cleaned using a PBS solution containing penicillin/streptomycin after the rats received intraperitoneal anesthesia with pentobarbital sodium (60 mg/kg). The ventricle tissues were cut into pieces of approximately 1 mm^3^ in size and softened with 0.125% trypsin (Gibco, USA). The cell suspension was combined with an equivalent amount of DMEM containing 10% FBS. After centrifuging the cell solution at 1000 × g for five minutes and filtering it through a 200-mesh filter, it was resuspended in 10% FBS DMEM. The cells were cultured at 37 °C with 5% CO₂ in an incubator. After three days, the culture medium was changed. The cell morphology was photographed using an inverted microscope. Vimentin antibody was used for the identification of atrial fibroblasts (27).

### Cell migration assay

The Transwell assay was utilized to evaluate the migratory capability of ventricular tissues. In 100 μl of DMEM devoid of FBS, cells were introduced into the upper chamber of the Transwell apparatus (8 μm, 1×10^5 cells/ml). The lower compartment was supplemented with 600 μl of DMEM containing 10% FBS. Cells in the upper section were carefully removed using a cotton swab after a 72-hour incubation period. The migrated cells were then labeled with 0.1% crystal violet solution. The quantified migrating cells were enumerated from five distinct fields using a light microscope (magnification, ×200) to calculate the mean sum of migrated cells (28).

### Cellular immunofluorescence

The methodology of immunofluorescence was executed as delineated in a previous study (29). Frozen slices of ventricular tissue from mice were acquired. Following three PBS rinses, DHE (S0063, Beyotime), α-SMA (ab7817, Abcam, UK), and Vimentin (ab92547, Abcam, UK) were given. After the cell nuclei were stained with DAPI, an inverted microscope (IX51, Olympus, Japan) was used for observation. Following the manufacturer’s instructions, Vimentin, α-SMA, and DHE were applied.

### Intracellular ROS level evaluation

DCFH-DA (1:1000) was applied to rat ventricular tissues for 30 min at 37 °C in the dark. Following that, the samples underwent three PBS washes. A microplate reader (IX71, Olympus, Japan) was used to measure the levels of intracellular ROS at 485 nm for excitation and 535 nm for emission (27).

### Measurement of oxidative stress indicators

Prior to examining oxidative stress biomarkers, including MDA (S0131S, Beyotime, Shanghai, China), SOD (S0109, Beyotime), and CAT (S0051, Beyotime) activities using commercially available kits, the ventricular tissues were homogenized (10%, w/v) to evaluate oxidative stress markers (27).

### RT-qPCR

We extracted the total RNA sample from ventricular tissues using a commercially available TRIzol reagent (Invitrogen, USA). Then, we carried out a reverse transcription reaction to convert complementary DNA (cDNA) from the total RNA. We performed an RT-qPCR reaction to amplify mRNA using an ABI Prism 7700 Real-Time PCR machine (Applied Biosystems, USA) and the SYBR Green reagent (TaKaRa, Japan). We measured the relative gene expression using the 2^-ΔΔCt^ procedure, where standardization with the housekeeping gene, GAPDH, was necessary (30). We designed primers using the online compatible NCBI Primer-BLAST Tool (https://www.ncbi.nlm.nih.gov/tools/primer-blast/). The primer nucleotide sequences applied in this research are presented in Table 1.

### Western blotting

We obtained the protein samples by cell lysis using a commercially available RIPA lysis buffer (Beyotime Biotechnology, Shanghai, China). We then increased the protein concentration using a commercially available BCA kit (Beyotime) and incorporated 40 μg of protein into each well. To denature the protein samples, we mixed them with a loading buffer from Beyotime and boiled the mixture in a water bath for three minutes. Electrophoresis was then performed for 30 minutes at 80 V, followed by 1 to 2 hours at 120 V, until the bromophenol blue reached the separation gel. After that, to transfer the protein onto the membrane, we placed it in the ice bath at 300 mA for 60 min. Before the membranes were sealed overnight at 4 °C or inactivated for an hour at room temperature, they were carefully cleaned using a washing solution for 1-2 min. Following this, the membranes were treated with primary antibodies against SIRT1 (1:500, sc-74465, mouse monoclonal, Santa Cruz), Nrf2 (1:500, ab92946, rabbit polyclonal, Abcam), HO-1 (1:500, ab305290, mouse monoclonal, Abcam), and GAPDH (1:1000, ab9485, rabbit polyclonal, Abcam) on a shaking table for one hour at ambient temperature. Before and after a one-hour incubation with the secondary antibody at 20 °C, the membranes were washed three times for ten minutes each with washing solution. Following the addition of the membranes to the developing solution, they were seen using a chemiluminescence imaging analysis device (Gel Doc XR, Bio-Rad) (27).

### Statistical analysis

All results were presented using the mean ± standard deviation (SD) from at least three studies. Comprehensive statistical analyses were conducted using the well-known software GraphPad Prism 9.0. Comprehensive statistical analyses were conducted using the well-known software GraphPad Prism 9.0. The *post hoc* Tukey test and one-way ANOVA were used to inspect group differences. *P*-values below 0.05 are regarded as statistically significant.

## Results

### Experimental evaluation of safety and physiological features in mice

For 28 consecutive days, mice were gavaged with SAL at 50 mg/kg/day. Blood urea nitrogen (BUN), creatinine, aspartate aminotransferase (AST), alanine aminotransferase (ALT), and other markers of hepatic and renal function were assessed in mice that received PBS and those that received SAL. The saline and SAL-treated mice showed no harmful effects on kidney and liver functions (Figure 1B and C). Furthermore, no adverse effects on the kidney or liver were detected with HE staining (Figure 1D). SAL was administered to mice by gavage (50 mg/kg/day) two hours prior to subcutaneous Ang II (1500 ng/kg/min) infusion for 28 days. Blood pressure was measured using the tail-cuff technique on days 0, 7, 14, 21, and 28. The findings indicated that SAL therapy reduced the rise in Ang II-infused SBP and DBP in mice (Figures 1E and F).

### Salidroside inhibits Ang II-infused cardiac dysfunction in mice

**Figure 2:**
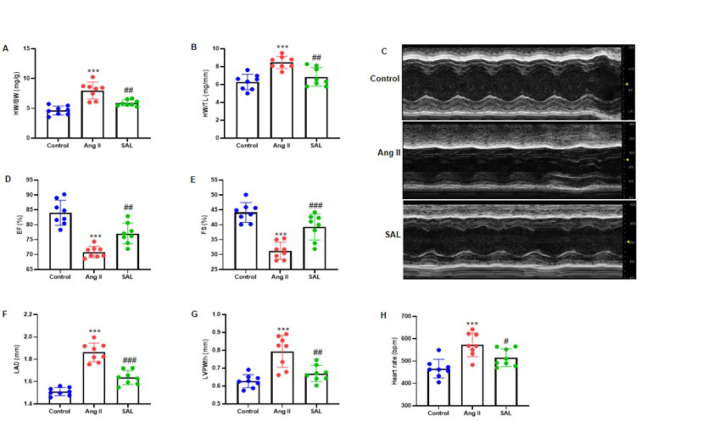
Salidroside prevents Ang II-induced cardiac dysfunction in mice

SAL treatment did not significantly inhibit the Ang II-infused elevate in the HW/BW and HW/TL ratios (Figures 2A and B). M-mode echocardiography was performed at 28 days after Ang II induction in mice gavaged with PBS or SAL. Echocardiography performed on day 28 revealed that administration of Ang II resulted in a notable augmentation of the left atrial diameter (LAD) in murine subjects. This phenomenon was subsequently suppressed following treatment with SAL (Figures 2C and F). SAL therapy included echocardiographic measures of cardiac functional factors, such as heart rate (bpm), left ventricular end-diastolic posterior wall thickness (LVPWth, mm), ejection fraction (EF, %), fractional shortening (FS, %), and LAD (mm). Our study revealed that SAL improved the EF and FS (Figure 2D and E). Additionally, SAL therapy attenuated Ang II-infused rises in LAD, LVPWth, and heart rate (Figure 2F-H).

### Salidroside attenuates Ang II-infused ventricular fibrosis

**Figure 3:**
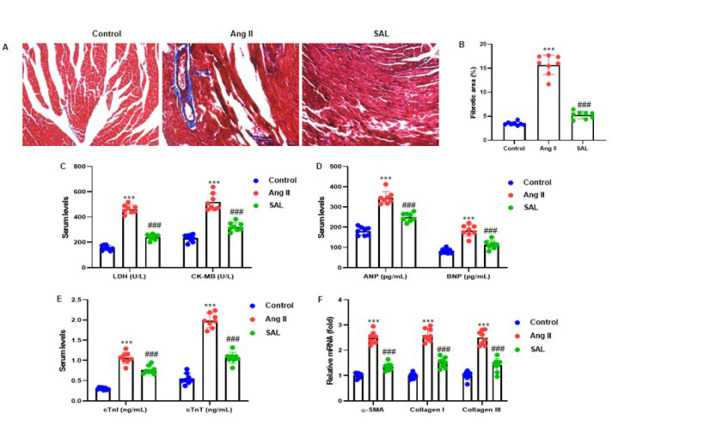
Salidroside suppresses Ang II-induced rat ventricular fibrosis

The implications of SAL on Ang II-induced ventricular fibrosis in murine models were rigorously examined. Cardiac fibrosis was quantitatively assessed through Masson trichrome staining of ventricular myocardial tissue, revealing that Ang II infusion markedly augmented the fibrotic area. SAL administration exhibited the potential to mitigate this fibrotic expansion (Figures 3A and B). Atrial natriuretic peptide (ANP), brain natriuretic peptide (BNP), cardiac troponin I (cTnI), cardiac troponin T (cTnT), creatine kinase isoenzyme (CK)-MB, and lactate dehydrogenase (LDH) were all measured in serum. The results elucidated that SAL significantly mitigated the Ang II-induced elevations in serum concentrations of CK-MB, LDH, ANP, BNP, cTnI, and cTnT (Figure 3C-E). The mRNA expression levels of α-smooth muscle actin (α-SMA), collagen I (COL1A1), and collagen III (COL3A1) were assessed using RT-qPCR techniques. It was noted that the administration of Ang II incited an up-regulation in the expression levels of α-SMA, collagen I, and collagen III. In contrast, SAL treatment markedly reduced these elevations (Figure 3F).

### Salidroside suppresses Ang II-infused rat atrial fibroblast migration

**Figure 4:**
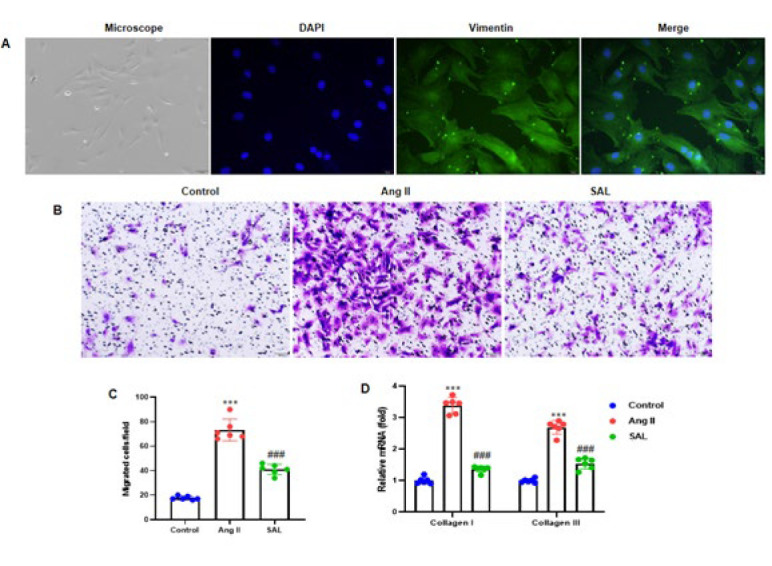
Salidroside inhibits the migration of rat atrial fibroblasts induced by Ang II

Primary cardiac atrial fibroblasts obtained from neonatal SD rats were identified using a vimentin antibody (Figure 4A). After a 2-hr pretreatment with 50 μM SAL, atrial fibroblasts were incubated with 1 μM Ang II for 48 hr. The results showed that SAL therapy potentially attenuated atrial fibroblasts stimulated by Ang II injection (Figure 4B). Transwell assays were used to investigate cell migration. By calculating how many migratory cells there were in each field across six randomly chosen areas, we were able to quantify cell migration and found that SAL could have lessened the increase in moved cells brought on by Ang II (Figure 4C). To assess the mRNA expression of Collagen I and Collagen III, we performed RT-qPCR analysis. We observed that Ang II infusion elevated Collagen I and Collagen III expression, while SAL treatment evidently reduced their expression (Figure 4D).

### Salidroside inhibits Ang II-infused atrial fibroblast differentiation

**Figure 5:**
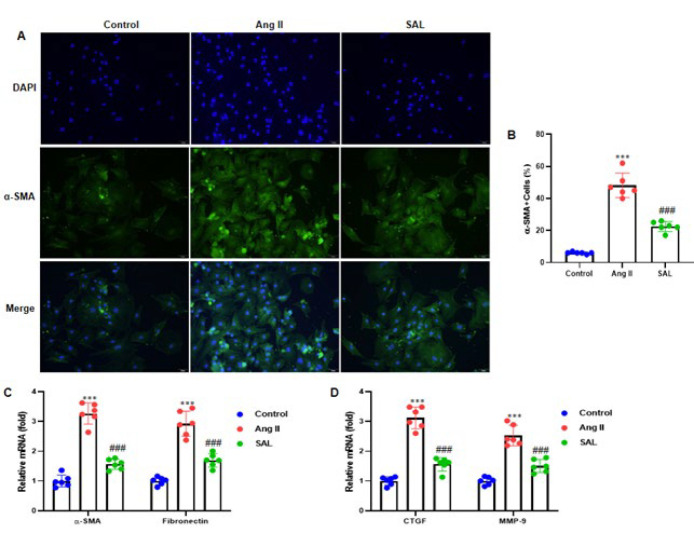
Salidroside attenuates Ang II-induced rat atrial fibroblast diﬀerentiation

Atrial fibroblasts were stained with α-SMA antibody to determine the differentiation of atrial fibroblasts. We observed that SAL therapy reduced the differentiation of atrial fibroblasts, which was increased by Ang II induction (Figure 5A and B). The RT-qPCR analyses were conducted to determine the mRNA expression of fibroblast differentiation-related genes, such as α-SMA, fibronectin, CTGF, and MMP-9. SAL therapy decreased the Ang II-infused rise in the α-SMA (ACTA2), fibronectin, CTGF, and MMP-9’s mRNA expression (Figures 5C and D).

### Salidroside activates SIRT1 to mediate its inhibitory impacts on Ang II-infused atrial fibroblast migration

**Figure 6:**
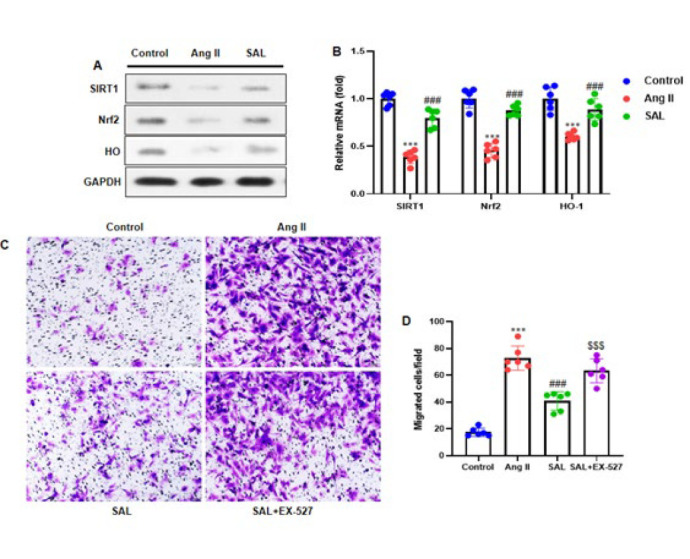
Activation of SIRT1 by salidroside mediates its inhibitory effects on Ang II-induced migration of rat atrial fibroblasts

Western blot examination was conducted to investigate the potential effect of SAL on SIRT1, Nrf2, and HO-1 expression in atrial fibroblasts. Ang II injection potentially inhibited the SIRT1, Nrf2, and HO-1 expression in atrial fibroblasts, elevated by SAL treatment (Figures 6A and B). Next, the SIRT1 inhibitor EX-527 (10 μM) was pretreated for two hours on atrial fibroblasts. Following this, the fibroblasts were incubated for two hours with 50 µM SAL and then exposed to Ang II (1 μM) for 48 hr. The Transwell test was conducted to evaluate the ability of atrial fibroblasts to migrate. The study outcomes indicated that Ang II infusion improved the number of atrial fibroblasts, inhibited by SAL therapy, and the SIRT1 inhibitor EX527 significantly elevated the migration ability (Figures 6C and D).

### Salidroside activates SIRT1 to mediate its inhibitory effects on Ang II-infused intracellular ROS production and oxidative stress in atrial fibroblasts

**Figure 7:**
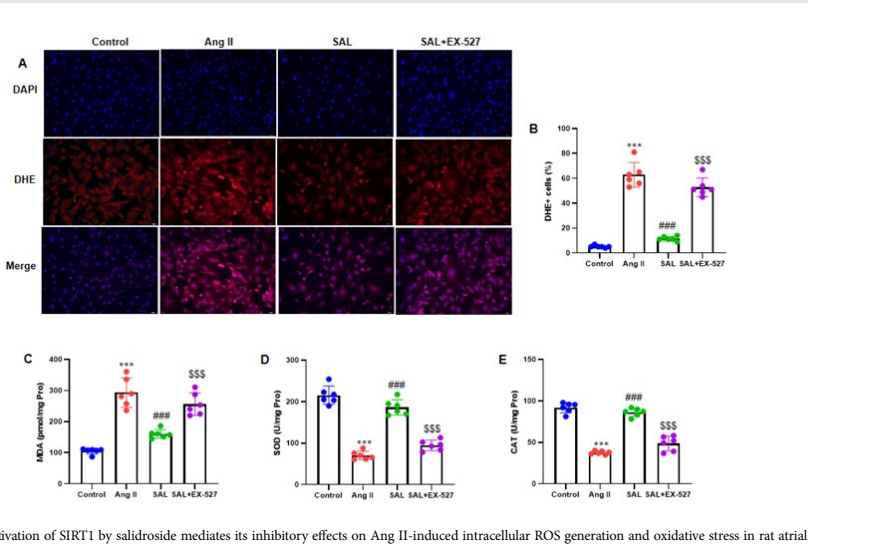
Activation of SIRT1 by salidroside mediates its inhibitory eﬀects on Ang II-induced intracellular ROS generation and oxidative stress in rat atrial fibroblasts

DHE labeling revealed a considerable increase in ROS production in atrial fibroblasts following Ang II treatment. Furthermore, the SAL+EX-527 pretreated group exhibited a substantial decrease in DHE-positive cells (*P*<0.001) in comparison to the Ang II group (Figures 7A and B). Among the oxidative stress indicators analyzed in the atrial fibroblast lysate were malondialdehyde (MDA), superoxide dismutase (SOD), and catalase (CAT). The results demonstrated that SAL therapy potentially reduced MDA activity while significantly increasing SOD and CAT activity. On the other hand, the SAL+EX-527 treatment reversed their activities (all *P*<0.001) (Figure 7C-E).

## Discussion

The current investigation explored the prospective role of SAL in the context of Ang II-infused myocardial fibrosis. The results indicated that SAL exerted no negative impacts on liver or kidney tissues while significantly attenuating systolic and diastolic blood pressure in murine models subjected to Ang II stimulation. SAL administration appeared to mitigate Ang II-infused cardiac dysfunction, ventricular fibrosis, and the migration and differentiation of rat atrial fibroblasts. Additionally, SAL was found to activate SIRT1, thereby mediating its inhibitory impacts on Ang II-infused atrial fibroblast migration, intracellular reactive oxygen species generation, and oxidative stress within atrial fibroblasts. Our results suggested that SAL may confer protective effects against Ang II-infused myocardial fibrosis.

Myocardial fibrosis is a cardiac condition characterized by improper repair of damaged or inflamed heart tissue. The primary pathological symptoms of structural cardiac disease are fibrotic and proliferative modifications in muscle tissue (31). The mechanism of myocardial fibrosis involves multiple complex interactions, including inflammation, oxidative stress, fibroblasts, extracellular matrix, and cytokines. The exact mechanism of myocardial fibrosis is currently unclear, and the clinical prognosis is poor. Consistent with the early investigations (32, 33), the present study showed the possible role of SAL in improving myocardial fibrosis in Ang II-induced mice. 

A recent study showed that traditional Chinese medicine is essential in preventing and treating myocardial infarction. It has significant effects and broad prospects in fibrosis. Recent research indicates that SAL has anti-inflammatory, anti-oxidant, antifibrotic, and antiarrhythmic impacts (34). SAL is the primary active substance in the traditional Chinese medicine *Rhodiola rosea* and has various therapeutic effects on systemic lesions. Studies have shown that it can inhibit fibrosis in multiple organs (35, 36). Cardiac fibroblasts are critical cells involved in cardiac fibrosis and are mainly responsible for the homeostasis of the extracellular matrix (37). They can increase, differentiate, and produce extracellular matrix proteins (38). However, in our research, we have used the Transwell assay to evaluate tissue migration ability. The migration rate of atrial fibroblasts was elevated remarkably in the Ang ll group compared to the control group. Consistent with previous studies (39), SAL treatment potentially reduced the migration ability of atrial fibroblasts infused by Ang II.

The mechanism of myocardial fibrosis is very complex. The currently recognized mechanism is RAS activation, which leads to excessive secretion and abnormal deposition of collagen fibrin as its primary mechanism. Previous research has reported that fibroblasts proliferate in the myocardium, and cardiac fibroblasts differentiate into α-SMA myofibroblasts, leading to the deposition of extracellular matrix collagen and abnormal collagen secretion (40) and ultimately promoting cardiac fibrosis. Collagen type I functions primarily as the principal structural framework, whereas Collagen type III governs elasticity. Collagen type I was observed to be predominant in rats with myocardial infarction, reducing ventricular wall elasticity. Following SAL treatment, there was a decrease in fibrosis area, indicating a partial restoration of ventricular compensatory capacity. These results provide compelling evidence of the potential of SAL to reverse myocardial fibrosis (32). In the present investigation, we examined the expression levels of genes linked to fibroblasts such as α-SMA, Collagen I, and Collagen III. Ang II infusion improved the α-SMA, Collagen I, and Collagen III levels. At the same time, SAL treatment significantly reduced their expression (Figure 3F). These results demonstrated that SAL treatment improved myocardial fibrosis, which is consistent with the early study.

Recent research findings have indicated a significant correlation between myocardial fibrosis and oxidative stress (41). The ROS function as secondary messengers within cells, playing a vigorous act in cell proliferation, differentiation, and apoptosis (42). The oxidative stress induced by ROS is closely linked to various cardiovascular conditions, such as heart failure, diabetic heart disease, myocardial ischemia/reperfusion injury, and myocardial fibrosis (43). The proliferation and differentiation of atrial fibroblasts are associated with higher ROS production, which enhances cardiac fibroblasts’ transformation into myofibroblasts and promotes myocardial fibrosis after myocardial infarction (44). The current work used DCFH-DA to detect intracellular ROS and the expression of oxidative stress markers such as MDA, SOD, and CAT. Our study demonstrated that SAL therapy potentially reduced ROS content and MDA activity while significantly increasing SOD and CAT.

SIRT1 belongs to the sirtuin family. Activation of SIRT1 hinders signaling pathways associated with oxidative stress, reduces the inflammatory mediators’ expression, and suppresses inflammation and tissue fibrosis (45). SIRT1 has been shown to exert a potent cardioprotective effect in cardiovascular disorders, and thus, pharmacological stimulation of SIRT1 may act as a novel therapeutic strategy to prevent myocardial fibrosis (46). However, the exact activation mechanism of SIRT1 is unknown. Our present study showed that SAL activates the SIRT1 protein. According to a recent study, when human umbilical vein endothelial cells (HUVECs) are exposed to oxidized low-density lipoprotein, SAL can enhance SIRT1 protein expression and decrease ROS production, thereby inhibiting oxidative stress, improving mitochondrial function and slowing down the pathological process of atherosclerosis (47). In addition, a previous study reported that SIRT1 increases anti-oxidant defenses and defends against Ang II-infused oxidative stress by activating Nrf2 (14). The SIRT1/Nrf2 pathway is influenced by Ang II signaling and works as a balance mechanism, enhancing anti-oxidant responses while suppressing inflammation (14, 15). However, our study showed that Ang II injection potentially inhibited the SIRT1, Nrf2, and HO-1 expression, which was elevated by SAL treatment (Figures 6A and B). These results indicated that SAL protects against myocardial fibrosis by activating the SIRT1-Nrf2 pathway.

There exist multiple constraints within our study. The sample size used for the investigation was not estimated using the power calculation. The power calculation will be carried out in future research to assess the study’s sample size. The *in vivo* investigations to evaluate the preventive impact of SAL against Ang II-infused cardiac fibrosis and its underlying mechanism did not involve female mice. The current study did not include a group of mice that received only SAL. Future studies will evaluate the effects on mice. The effectiveness of SAL was not assessed in a manner that varied by dosage. The effectiveness of using different dosages of the SAL will be assessed in a future study. Neither preclinical nor clinical settings have validated the findings of the study. In both preclinical and clinical settings, the study outcomes will be verified. Furthermore, further scrutiny is warranted to elucidate the causal mechanism responsible for SAL’s protective properties against Ang II-infused myocardial fibrosis. Nevertheless, the investigation offers substantial proof of SAL’s protective attributes against myocardial fibrosis.

(A) Chemical structure of salidroside. Hepatic and renal function serum indicators, including (B) ALT, AST, (C) creatinine and BUN, were measured in PBS solution and salidroside-gavaged mice. (D) Hepatic and renal tissues of mice were stained with HE (magnification 200×). The tail-cuff method measured blood pressure on days 0, 7, 14, 21, and 28. Salidroside decreased (E) SBP and (F) DBP in Ang II-infused mice. Data are presented as the mean ± SD (n=8 in each group). Data are presented as the mean ± SD (n=8 in each group). SAL: salidroside; Ang II: angiotensin II; ALT: alanine transaminase; AST: aspartate transaminase; BUN: blood urea nitrogen; SBP: systolic blood pressure; DBP: diastolic blood pressure. ***P<0.001 vs Control group; #*P*<0.05, ##*P*<0.01, ###*P*<0.001 vs Ang II group.

**Table 1:** Sequences of primers for mouse and rat genes used in this current study

Genes	Forward primer (5′-3′)	Reverse primer (5′-3′)
Mouse α-SMA	GTCCCAGACATCAGGGAGTAA	TCGGATACTTCAGCGTCAGGA
Mouse Collagen I	GAGTACTGGATCGACCCTAACCA	GACGGCTGAGTAGGGAACACA
Mouse Collagen III	TCCCCTGGAATCTGTGAATC	TGAGTCGAATTGGGGAGAAT
Rat Collagen I	CGGTGGTTATGACTTCAGCTTC	AGAGGGCTGAGTGGGGAAC
Rat Collagen III	CGGGCAAGAATGGAGCAAAG	ACCAGGGAAACCCATGACAC
Rat α-SMA	CTATTCCTTCGTGACTACT	ATGCTGTTATAGGTGGTT
Rat Fibronectin	TGACGAGGACACGGCAGAGC	AGGAATGGCTGTGGACTGGACTC
Rat CTGF	GTGTGCACTGCCAAAGATG	TCGGTAGGCAGCTAGGGC
Rat MMP-9	CCCTGCGTATTTCCATTCATC	ACCCCACTTCTTGTCAGCGTC
Mouse GAPDH	ACTCCACTCACGGCAAATTC	TCTCCATGGTGGTGAAGACA
Rat GAPDH	GACATGCCGCCTGGAGAAAC	AGCCCAGGATGCCCTTTAGT

α-SMA, α-smooth muscle actin; CTGF: connective tissue growth factor; MMP-9: matrix metalloproteinases-9; GAPDH: Glyceraldehyde-3-phosphate dehydrogenase

(A, B) HW/BW and HW/TL ratios in mice in the control, Ang II, and SAL groups. (C) Representative M-mode echocardiography images are shown. Echocardiographic measurements of cardiac functional parameters are as follows: (D) ejection fraction (EF, %); (E) fractional shortening (FS, %); (F) left atrial diameter (LAD, mm); (G) left ventricular end-systolic posterior wall thickness (LVPWth, mm); and (H) heart rate (bpm). Data are the mean ± SD (n=8 in each group). HW: heart weight; BW: body weight; TL: tibia length; IVS: interventricular septum; LV: left ventricle; ***P<0.001 vs Control group; #*P*<0.05, ##*P*<0.01, ###*P*<0.001 vs Ang II group.

(A) Ventricular fibrosis was evaluated by staining atrial fibroblasts with Masson trichrome (blue area, and magnification 200×). (B) Atrial fibrosis was quantified by calculating the percentage of the fibrotic area. Colorimetry and ELISA were performed to determine the serum levels of (C) LDH (U/L), CK-MB (U/L), (D) ANP (pg/ml), BNP (pg/ml), (E) cTnI (ng/ml), and cTnT (ng/ml) in mice. (F) Expression of α-SMA, Collagen I (COL1A1), and Collagen III (COL3A1) was analyzed using RT-qPCR. Data are shown as the mean ± SD (n = 8 in each group). ***P<0.001 vs the control group; ###*P*<0.001 vs the Ang II group. α-SMA: α-smooth muscle actin.

(A) Primary rat atrial fibroblasts were isolated from neonatal SD rats and identified by Vimentin antibody. Representative images of atrial fibroblasts under an inverted microscope (200×) or immunofluorescence (400×) are shown. (B) Atrial fibroblasts were pretreated with salidroside at 50 μM for 2 hr and incubated with 1 μM Ang II for 48 hr. (C) The number of migrated cells per field was quantified from 6 random fields. (D) RT-qPCR was conducted to determine the mRNA expression of Collagen I (COL1A1) and Collagen III (COL3A1). GAPDH served as an internal control. Data are shown as the mean ± SD (n=6 per group) from three independent experiments. **P<0.01, ***P<0.001 vs the control group; #*P*<0.05, ##*P*<0.01, ###*P*<0.001 vs Ang II group.

(A) Atrial fibroblasts were stained with α-SMA antibody (200×). (B) Quantitative analysis of α-SMA-positive cells normalized to DAPI-stained cells. Salidroside attenuated the Ang II-induced increase in the mRNA expression of (C) α-SMA (ACTA2), fibronectin, (D) CTGF and MMP-9. GAPDH served as an internal control. Data are shown as the mean ± SD (n=6 per group). CTGF: connective tissue growth factor; MMP-9: matrix metalloproteinases-9. ***P<0.001 vs the control group; ###*P*<0.001 vs the Ang II group.

(A) Western blotting was performed on atrial fibroblasts, and representative bands of SIRT1, Nrf2, and HO-1 are shown. (B) Quantitative analysis of protein bands that are normalized to GAPDH. The atrial fibroblasts were pretreated with the SIRT1 inhibitor EX-527 (10 μM) for two hours, followed by incubation with 50 µM salidroside for two hours and further incubation with Ang II (1 μM) for 48 hr. (C) The cell migrative capability of atrial fibroblasts was determined by Transwell assay. (D) Quantitative analysis of the average number of migrated tissues in each field. Data are shown as the mean ± SD (n=6 per group). ***P<0.001 vs control group; ###*P*<0.001 vs Ang II group; $$$ *P*<0.001 vs SAL group.

(A) Intracellular ROS generation in atrial fibroblasts was evaluated by staining with DHE (200×). (B) Quantification of intracellular ROS levels by calculating DHE-positive cells normalized to DAPI-stained cells. The oxidative stress markers were measured in the cell lysate of atrial fibroblasts for (C) MDA, (D) SOD, and (E) CAT. Data are shown as the mean ± SD (n=6 per group). DHE: dihydroethidium; MDA: malondialdehyde; SOD: superoxide dismutase; CAT: catalase. ***P<0.001 vs control group; ###*P*<0.001 vs Ang II group; $$$ *P*<0.001 vs SAL group.

## Conclusion

The current investigation assessed the possible protective impacts of SAL on myocardial fibrosis infused by Ang II in a murine model. The study findings showed that SAL intervention significantly mitigated Ang II-infused ventricular fibrosis by up-regulating the SIRT1-Nrf2 signaling pathway. Validating these findings in preclinical and clinical settings is essential.

## Data Availability

Due to confidentiality issues, the datasets generated and/or analyzed during the current work are not publicly available but are available from the corresponding author upon reasonable request.

## Declaration

We have not used any AI tools or technologies to prepare this manuscript.
